# A Non-sulfided flower-like Ni-PTA Catalyst that Enhances the Hydrotreatment Efficiency of Plant Oil to Produce Green Diesel

**DOI:** 10.1038/srep15576

**Published:** 2015-10-27

**Authors:** Jing Liu, Pan Chen, Lihong Deng, Jing He, Luying Wang, Long Rong, Jiandu Lei

**Affiliations:** 1 Beijing Key Laboratory of Lignocellulosic Chemistry, Beijing Forestry University, Beijing 100083, P. R. China; 2 Key Laboratory for Biomechanics and Mechanobiology of Ministry of Education, School of Biological Science and Medical Engineering, Beihang University, Beijing 100191, P. R. China

## Abstract

The development of a novel non-sulfided catalyst with high activity for the hydrotreatment processing of plant oils, is of high interest as a way to improve the efficient production of renewable diesel. To attempt to develop such a catalyst, we first synthesized a high activity flower-like Ni-PTA catalyst used in the hydrotreatment processes of plant oils. The obtained catalyst was characterized with SEM, EDX, HRTEM, BET, XRD, H_2_-TPR, XPS and TGA. A probable formation mechanism of flower-like Ni(OH)_2_ is proposed on the basis of a range of contrasting experiments. The results of GC showed that the conversion yield of Jatropha oil was 98.95%, and the selectivity of C11-C18 alkanes was 70.93% at 360 °C, 3 MPa, and 15 h^−1^. The activity of this flower-like Ni-PTA catalyst was more than 15 times higher than those of the conventional Ni-PTA/Al_2_O_3_ catalysts. Additionally, the flower-like Ni-PTA catalyst exhibited good stability during the process of plant oil hydrotreatment.

In recent years, vegetable oils have been investigated as a way to provide a renewable source for the production of biodiesel by catalytic hydrogenation^1^. Hydrocracking of plant oil leads to the production of straight chain alkanes consisting of 11–20 carbons, known as green diesel, or renewable diesel^2^. Conventional hydrotreatment catalysts such as NiMo, NiW, or Pt supported on zeolite (ZSM-5, SAPO-11) or/and γ-Al_2_O_3_ have been mostly used for this process^3^,^4^,^5^,^6^,^7^. The nickel-based catalysts are usually sulfided to retain their active form^8^, however, these catalysts are very susceptible to poisoning by atmospheric sulfur^9^. Sulfuration also may cause sulfur dioxide emissions, corrosion, and sulfur residues in the products; plant oils are usually free of sulfur compounds. Although noble metal catalysts can be used without sulfuration, their application might be limited because of their high cost. Therefore, it is very important to find a high activity catalyst that does not require sulfidizing and that is suitable for large-scale production.

Several non-sulfided hydrotreatment catalysts have been recently reported. Márton Krár *et al*^10^. has investigated the catalytic conversion of sunflower oil with non-presulphided CoMo/Al_2_O_3_ and NiMo/Al_2_O_3_ catalysts. Both of these non-presulphided commercial catalysts exhibit high deoxygenation activity and isomerization activity. However, the triglyceride conversion (25.1–30.5%) and yield (24.2–24.6%) are too low. Xinghua Zhang *et al*^11^. developed non-sulfided Ni/HZSM and Ni/Al_2_O_3_ catalysts for hydrotreatments using phenol as a model compound. Both catalysts worked but were not ideal. Conversion with Ni/Al_2_O_3_ catalysts was only 20.9%. The Ni/HZSM conversion reached 91.8%, but Ni/HZSM has a very small pore size (2.2 nm). Since triglyceride is a macromolecule, the use of a catalyst with a small pore diameter would decrease catalytic activity by restricting the access of triglyceride to the catalytic sites^12^. Previously, we prepared a non-sulfided Al_2_O_3_ supported rare metal^13^, but the product contained few iso-paraffins and the product oil had insufficient cold flow properties.

In order to design and develop a non-sulfided and high activity catalyst with suitable morphology and structure for the hydrotreatment of plant oil, we investigated the use of flower-like nickel oxide (NiO), which has been used extensively for catalysis^14^,^15^. The pore diameter of flower-like NiO is about ten times larger than that of Ni/Al_2_O_3_ 16,17, which would readily allow the diffusion of bulky triglyceride molecules and prevent pore blockage. In addition, Heteropoly Acids (HPA), such as phosphotungstic acid (PTA), possess unique physicochemical properties, and their structural mobility and multifunctionality could promote catalysis^18^. However, the low surface area of PTA (<10 m^2^/g) limits practical applications^19^. Thus, direct immobilization of PTA on the surface of flower-like NiO could increase the contact area between the acid center and reactants to improve the catalytic activity. Moreover, appropriate amounts of macropores would favor the impregnation of large-size PTA molecules. Our previous studies have found that phosphotungstic acid (PTA) combined with Ni/Al_2_O_3_ can be used as bifunctional catalysts and is effective for the hydrocracking of Jatropha oil^20^. Therefore, the flower-like Ni-PTA catalyst can be obtained by flower-like NiO supported PTA by the impregnating method. As a bimetallic catalyst^21^ containing solid acid with flower-like morphology and porous structure, flower-like Ni-PTA catalyst will potentially exhibit superior performance in hydrotreatment of plant oil without sulfuration.

This is the first report of synthesis of a non-sulfided nickel and phosphotungstic acid (Ni-PTA) catalyst with flower-like morphology and macropores for the hydrotreatment of plant oil, using the non-edible plant oil (Jatropha oil) as the feedstock. The flower-like porous NiO was first synthesized using the hydrothermal method. Then, the flower-like Ni-PTA catalyst was obtained by impregnation of HPA into the flower-like NiO. Secondly, flower-like NiO and flower-like Ni-PTA were characterized by SEM, TEM, EDX, BET, and XRD. Finally, the conversion of Jatropha oil and the selectivity of C11-C18 of the flower-like Ni-PTA catalysts were determined.

## Methods

### Synthesis of flower-like Ni-PTA catalyst

**Figure 1:**
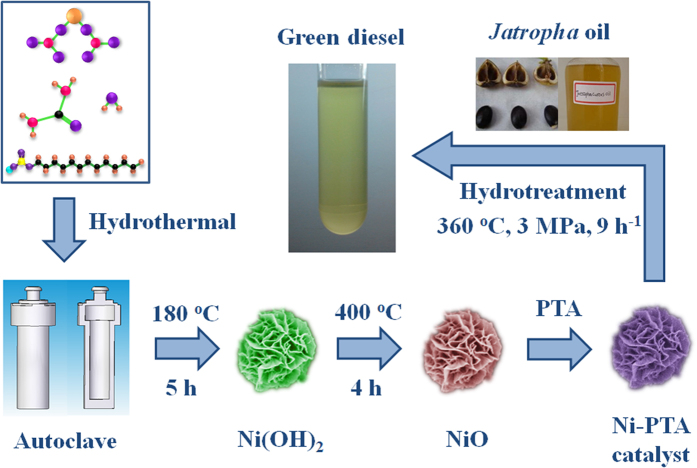
Schematic diagram of synthesis of flower-like Ni-PTA catalyst and its catalytic performance in the hydrotreatment of Jatropha oil (autoclave was drawn by Pan Chen).

A flower-like Ni-PTA catalyst was prepared by a hydrothermal method. The synthetic procedure is illustrated in Fig. 1. Details of the representative reactions are as follows. First, 2 g of Ni(NO_3_)_2_ · 6H_2_O, 0.8 g of CO(NH_2_)_2_, and 1 g of SDS was dissolved in 40 mL distilled water. After the mixture was magnetically stirred for 15 min at room temperature, the solution was then transferred into a Teflon-lined stainless-steel autoclave of 100 mL capacity. After heating at 180 °C for 5 h, the tank was cooled down to room temperature naturally. The resultant green products were collected using a centrifuge, then washed three times with distilled water and absolute ethanol, and dried under vacuum at 60 °C for 2 h. Finally, the flower-like Ni(OH)_2_ was calcined in a muffle oven at 400 °C for 4 h. Next, the flower-like Ni-PTA catalyst was prepared by impregnanting flower-like NiO with a solution containing PTA (30 wt%)^22^. Impregnated samples were dried at 100 °C for 3 h and calcined at 200 °C for 3 h.

### Characterization of flower-like Ni-PTA catalyst

The morphology of the catalyst was characterized by scanning electron microscopy (SEM, S-3400N) and high-resolution transmission electron microscopy (HRTEM, JEOL JEM-2100F). HRTEM was conducted at an acceleration voltage of 200 kV to obtain a lattice resolution of 0.2 nm. Elemental mapping images of samples were taken with a CamScan Apollo300 scanning electron microscope (SEM) equipped with X-ray energy-dispersive (EDX) microanalyzer (OXFORD INCA). The specific surface areas were obtained from Brunauer-Emmett-Teller (BET) N_2_ adsorption-desorption isotherms, measured on a V-Sorb 2800 TP Surface Area and Pore Distribution Analyzer instrument, and the pore size distributions were estimated from the adsorption branch of the N_2_ isotherms by the Barrett-Joyner-Halenda (BJH) method. Wide-angle powder X-ray diffraction (XRD) patterns were measured using an 18 kW diffractometer (Bruker D8 Advance) with monochromated Cu Kα radiation. Samples were measured in the 2θ range from 10° to 80° (scan speed of 2° per minute). The temperature-programmed reduction with hydrogen (H_2_-TPR) was performed using a quartz micro reactor TP-5080 (Tianjing-Xianquan, China). About 50 mg of sample was pre-treated under He atmosphere at 300 °C for 60 min and then cooled down to 40 °C. After switching to a H_2_/He mixture (40 mL/min, 5 vol.%), H_2_-TPR was started at a heating rate of 10 °C/min up to 800 °C. The H_2_ consumption rate was monitored with a thermal conductivity detector (TCD) and its signal was transmitted to a personal computer. X-ray photoelectron spectroscopy (XPS) data were obtained using an ESCALab250 electron spectrometer from Thermo Scientific Corporation with monochromatic 150 W AlKα radiation. The base pressure was about 6.5 × 10^−10^ mbar. The binding energies were referenced to the C1s line at 284.8 eV from alkyl or adventious carbon. The coke formed on the spent catalyst was evaluated by Thermogravimetric analysis (TGA, SDT-Q600). The used catalyst was first heated from 30 °C to 550 °C at a heating rate of 30 °C/min in N_2_ using a flow of 100 ml/min, at a constant temperature of 550 °C for 15 min. It was then heated linearly at 30 °C/min to 900 °C in 100 ml/min O_2_. The weight loss of samples was processed by microcomputer.

### Catalytic properties

The hydrotreatment of Jatropha oil into green diesel was used to examine the performance of flower-like Ni-PTA catalyst. Experiments were performed in a fixed-bed reactor equipped with an electrical heating system. A detailed description of the apparatus is provided elsewhere^23^. The non-sulfided catalyst (0.6 g) was loaded into a stainless steel tubular reactor (1.2 cm I.D and 56 cm in length) and activated *in situ* prior to the experiments with H_2_ at 400 °C and 3 MPa for 3 h. The reaction conditions for the hydrotreatment experiment were as follows: temperature 360 °C, pressure 3 MPa, liquid hourly space velocity (LHSV) 15 h^−1^, and H_2_ to feed ratio of 1000 mL H_2_ gas/mL liquid feed.

The liquid products were withdrawn after stabilization of reaction conditions (6 h) in one-hour intervals and analyzed by off-line gas chromatography (GC) after separation of the water phase. Gaseous products and water were not further analyzed. Liquid products were analyzed using a GC-900C equipped with AT. SE-30 column (L = 30 m, d = 0.32 mm, tf = 0.5 μm) and detected by a flame ionization detector (FID). Helium was used as the carrier gas. The following temperature program was used: initial temperature at 60 °C for 2 min, heating at 15 °C/min to 210 °C, then at 8 °C/min to 270 °C, and finally at 5 °C/min to 285 °C, with a dwelling time of 5 min at 285 °C. Individual products were identified by GC standards. The conversion of Jatropha oil was calculated as:


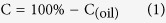


where C_(oil)_ is the concentrations of triglycerides (%) in the product oil, determined by GC analysis. The selectivity of C11-C18 alkanes was calculated as:


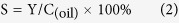


where Y is the yield of the C11-C18 alkane (%), determined by GC analysis, and C is the conversion of Jatropha oil (%), calculated with Eq. (1).

## Results and Discussion

### Catalyst characterization

**Figure 2:**
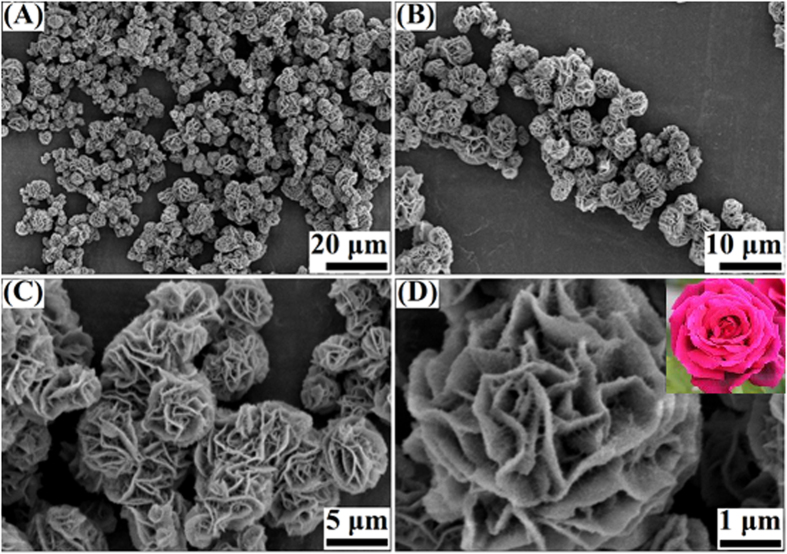
SEM images of flower-like Ni(OH)_2_ at different magnifications, inset of (**D**) shows a peony flower found in nature (photo was taken by Jiande Lei).

The morphology and size of the synthesized samples were characterized by SEM. The low-magnification SEM image (Fig. 2A,B) shows that the products consist of a high yield of fairly uniform flower-like architectures. The high-magnification SEM images (Fig. 2C,D) clearly show flower-like shapes having a size of ca. 5 μm with dozens of flake-like nanopetals that seem to grow from a centre. This morphology is similar to that of the peony flower (shown in the inset of Fig. 2D). Figure 2 shows a large quantity of flower microspheres. The porous structure may enable the triglyceride molecules to penetrate and be easily adsorbed onto the pore network matrices^24^.

**Figure 3:**
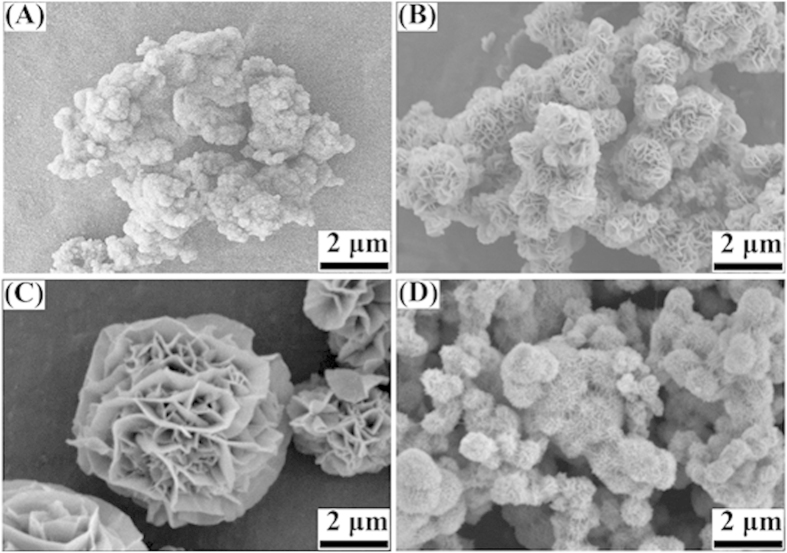
SEM images of the synthesized flower-like Ni(OH)_2_ with treatment temperatures of (**A**) 110 °C, (**B**) 120 °C, (**C**) 220 °C, and (**D**) 180 °C without SDS for 5 h.

In this study, the reaction temperature was found to have significant effects on the product morphology, when other reaction conditions were kept constant (5 h with SDS). At 110 °C monodispersed flower-like Ni(OH)_2_ structures were not obtained, and there existed many nanoparticles with a wide size distribution (Fig. 3A). When the temperature was increased to 120 °C, flower-like Ni(OH)_2_ architectures with a small size of ca. 0.8–1.5 μm formed (Fig. 3B). If the reaction temperature was higher than 120 °C, the size of flower-like Ni(OH)_2_ architectures increased with the temperature enhancement. When the reaction was maintained at 180 °C, there was no obvious change in the morphology of flower-like architectures beyond the obvious increase in particle size (ca. 5 μm, see Fig. 3). Compared to samples produced at 180 °C, the microstructure and size appeared identical to those produced at 220 °C (Fig. 3C).

SDS, an anionic surfactant, has been widely used as a surface modifier for the preparation of flower-like structures^25^,^26^. In order to investigate the effect of SDS on morphology, a control experiment was performed in the absence of SDS. Clusters of Ni(OH)_2_ with a much broader size distribution and lacking flower-like architecture were formed (Fig. 3D), suggesting that SDS plays an important role in the formation of the flower-like architecture. SDS physically absorbed on the (111) direction of Ni subunits can inhibit the growth in the (111) direction, resulting in the oriented growth of nanoflakes during the reaction process. Yingying Duan *et al*^27^. similarly reported that CuO nanoflowers were able to be assembled from primary helically arranged “nanoflakes” with the assistance of SDS as the structure-directing agent.

**Figure 4:**
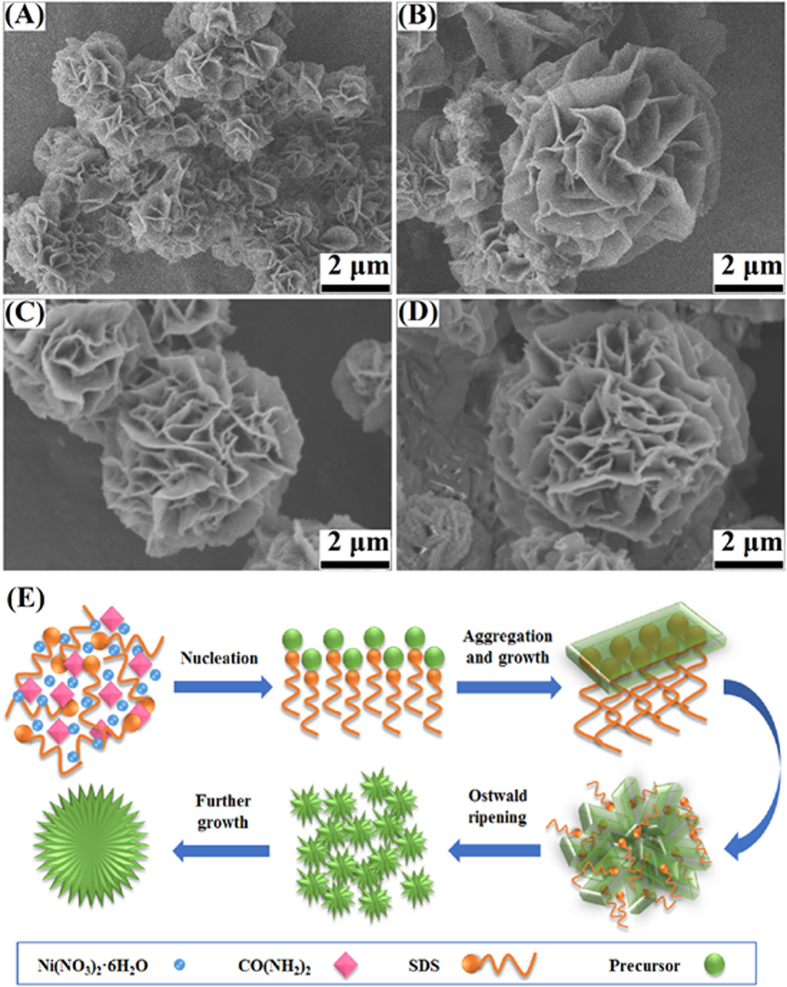
SEM images of the synthesized flower-like Ni(OH)_2_ with reaction time of (**A**) 1 h, (**B**) 3 h, (**C**) 5 h, and (**D**) 7 h at 180 °C; (**E**) Schematic illustration for the formation mechanism of flower-like Ni(OH)_2_ architectures (diagram was drawn by Jing Liu).

Reaction time was also found to play an important role in the formation process of the flower-like Ni(OH)_2_ architectures. Figure 4 shows the SEM images of samples obtained at 180 °C after 1 h, 3 h, 5 h, and 7 h, respectively. After 1 h of reaction (Fig. 4A), aggregates of microspheres with a larger size of ca. 1 μm were the predominant products and were composed of thick sheets. This was likely due to crystal splitting that can occur at the whole surface of the limited nuclei to form the flowerlike structure^28^. When the reaction time was prolonged to 3 h (Fig. 4B), the size of the 3-D structures increased gradually and the morphology became a flower-like structure with a network of nanosheets on their surfaces. The flower-shaped structures were not well-developed, as if they were in the initial stage of the growth. When the reaction time was extended to 5 h (Fig. 4C), the flower-like morphology gradually became more clearly defined. The special 3D flower-like morphology should provide sufficient space and open channels for triglyceride penetration and products of alkane rapid diffusion, which would be advantageous to hydrotreatment performance. With an extension of reaction time (7 h, Fig. 4D), the morphologies remained similar to those seen with 5 h of treatment, but the diameter of the particles increased to ca. 7 μm.

The slow crystal growth process evident from these findings may be due to the slow reaction. The relevant chemical reactions in the hydrothermal process can be written as follows^29^,^30^:


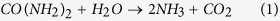



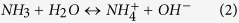



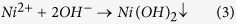


As shown in reaction (1), the thermal decomposition of urea occurred rapidly after being heated to 100 °C, followed by the release of NH_3_ and CO_2_. Aqueous ammonia plays an important role in the growth of Ni(OH)_2_ flower-like structures. It not only acts as an alkaline reagent to provide OH^−^ ions for the formation of Ni(OH)_2_, but also as a complexing reagent for the Ni^2+^ ions to form [Ni(NH_3_)_6_]^2+^. The formation of [Ni(NH_3_)_6_]^2+^ may control the reaction rate of Ni^2+^ ions with OH^−^ ions and thereby control the formation of Ni(OH)_2_ flower-like structures^31^,^32^. A slow reaction rate would cause the separation of nucleation and growth steps, crucial for high-quality crystal synthesis.

Our findings clearly indicated that the morphology evolution was a function of the hydrothermal reaction time. The process is illustrated in Fig. 4E. This process is consistent with the previously reported so-called two-stage growth process, which involves a fast nucleation of amorphous primary particles followed by a slow aggregation and crystallization of primary particles^33^. Jing Kong *et al*^34^. reported a similar progress in preparation of Ni flower-like architectures.

**Figure 5:**
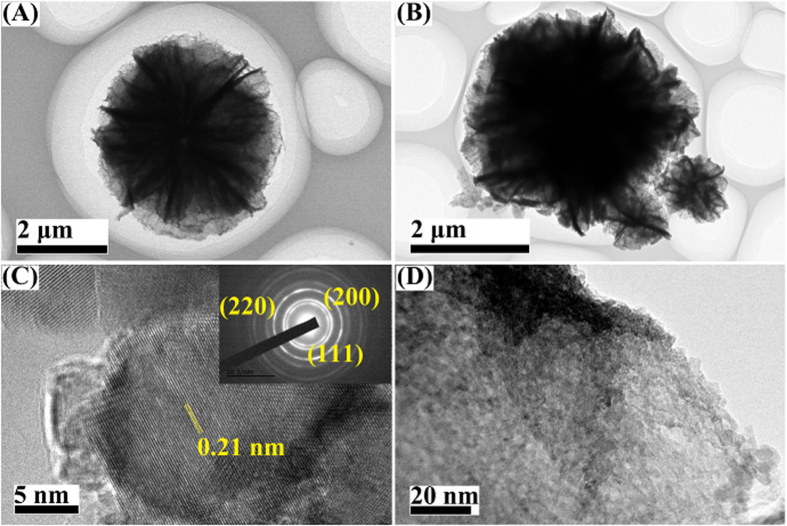
TEM images of flower-like NiO (**A**) and flower-like Ni-PTA catalyst (**B**). Image (**C**) is HRTEM image taken from the edge of flower-like NiO sheets and the corresponding SAED pattern. Image (**D**) is the detailed structures of flower-like Ni-PTA “petals”.

The morphologies and structures of samples were further characterized by TEM and HRTEM. As shown in Fig. 5A,B, the obtained flower-like structures have a solid interior core with nanoflakes growing on it, with the diameter of the samples being about 4 μm. The NiO was obtained by decomposition of the Ni(OH)_2_ at 400 °C for 4 h. The morphology of NiO is similar to that of the precursor, indicating that the morphology can be well preserved during phase transformation^35^. After loading PTA nanoparticles, the morphologies of the flower-like structure were mostly preserved, indicating that the flower-like architectures are very stable and the complex architectures are actually integrated^36^. Figure 5D shows detailed structures of the flower-like Ni-PTA catalyst “petals”. According to the HRTEM image (Fig. 5C), the lattice fringe spacing is about 0.21 nm, close to the *d*_111_ of cubic NiO. The corresponding SAED pattern is shown in the inset of Fig. 5C, and suggests that the NiO surface is of (111) orientation^37^. The lattice fringes and the diffraction rings matched the XRD peaks very well, which indicated that the building units are polycrystalline^38^.

**Figure 6:**
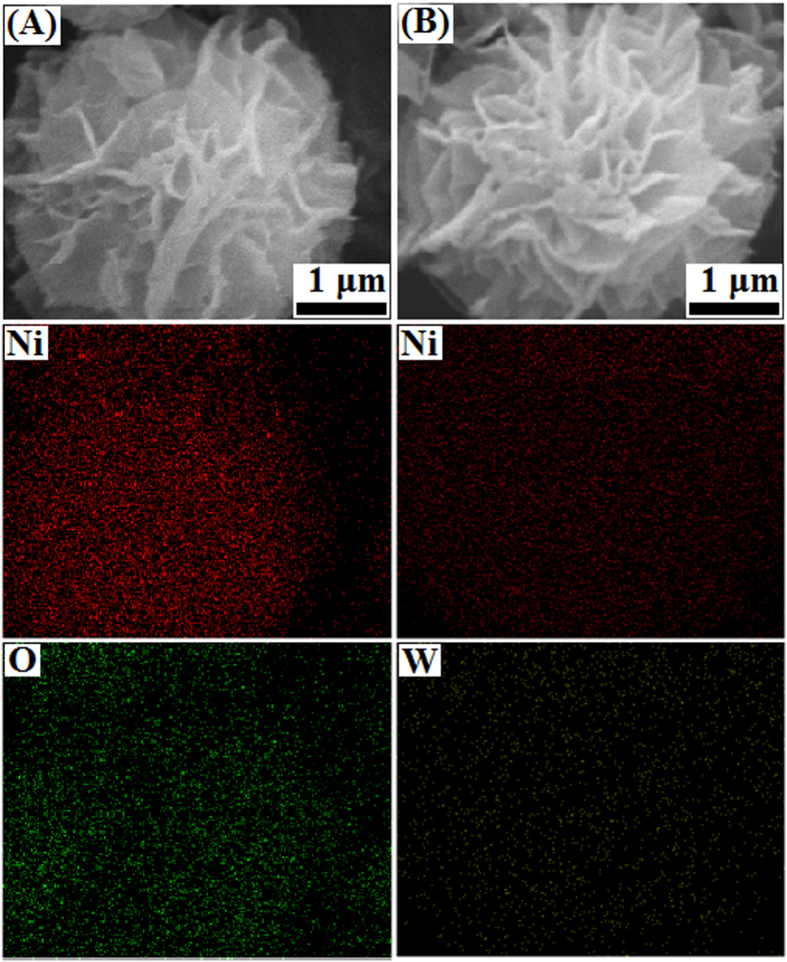
SEM-EDX elemental mappings for the flower-like NiO (**A**) and flower-like Ni-PTA catalyst (**B**).

The chemical composition of these microflowers was further determined by SEM-EDX (Fig. 6). Elemental analysis shown in Fig. 6A suggests that the flower-like NiO consists solely of Ni and O elements. However, significant differences in the atomic distribution of Ni and O were evident, and we found a [Ni]/[O] ratio of 0.72 and 0.51 for flower-like NiO and flower-like Ni-PTA catalyst, respectively. The SEM-EDX mapping of Fig. 6B revealed the surface distribution of Ni and W. It indicates that Ni and W were uniformly distributed in the flower-like Ni-PTA catalyst surfaces, and the corresponding elementary analysis revealed that the atomic ratio of Ni to W was 6.05:1. SEM, TEM, SEM-EDX results indicated that the flower-like Ni-PTA catalyst were successfully synthesized by hydrothermal and impregnation methods, and will potentially exhibit superior performance in hydrogenation catalytic activity.

**Figure 7:**
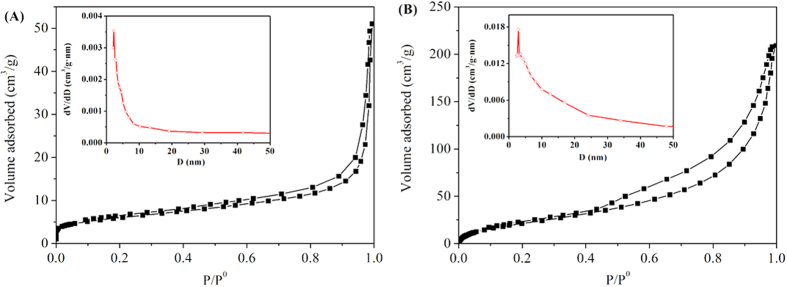
N_2_ adsorption-desorption isotherms and pore size distribution curves (inset) of flower-like Ni(OH)_2_ (**A**) and flower-like Ni-PTA catalyst (**B**).

The N_2_ adsorption-desorption isotherms and the corresponding BJH pore size distribution plots of the obtained flower-like product are shown in Fig. 7. According to the IUPAC classification^17^, the isotherms of the Ni(OH)_2_ and the Ni-PTA catalyst can be classified as type IV, with H3 type hysteresis behavior, a feature of mesoporous material^39^. Both of them have a distinct hysteresis loop observed in the range of 0.4–1.0 P/P_0_. The measured BET surface areas for the flower-like Ni(OH)_2_ and the flower-like Ni-PTA catalyst were 21.4 and 85.9 m^2^/g, respectively. Such an increase of specific surface areas are probable results because after calcining in high temperature, the impurities are oxidized, decomposed, or removed from the micropore, leading to pore formation. The pore size distribution graphs are displayed in the insets of Fig. 7, and the BJH median pore width for the flower-like Ni(OH)_2_ and the flower-like Ni-PTA catalyst were 2.03 nm and 2.85 nm, respectively. The average pore diameters were determined to be 20.5 nm and 12.6 nm, respectively, according to the BJH plots calculated from the N_2_ desorption isotherms of the two samples, and the corresponding BJH desorption cumulative volumes are 0.08 and 0.34 cm^3^/g, respectively. Taken together, these results indicate that our method yields a high degree of mesopore orientation in the flower-like Ni-PTA catalyst, with high surface area and porous characteristics. This morphology should allow a very short diffusion pathway for triglyceride molecules, leading to enhanced catalytic activity.

**Figure 8:**
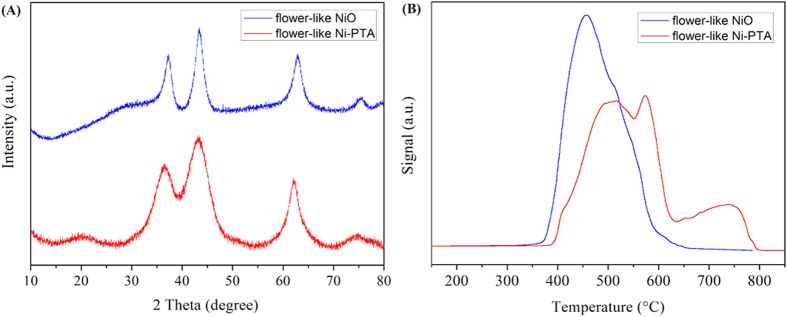
XRD (**A**) and H_2_-TPR (**B**) patterns of flower-like NiO and flower-like Ni-PTA catalyst.

The phase structure and purity of the products were examined by XRD, as shown in Fig. 8A. The wide angle XRD pattern exhibited typical diffraction peaks of cubic NiO phase (JCPDS 78–0643)^21^. The diffraction peaks corresponding to NiO appeared at 2θ = 37.28°, 43.28°, 62.88°, 75.28° and 79.48°, respectively, and can be readily indexed as (111), (200), (220), (311) and (222) crystal planes^24^. Since no other impurity peaks were detected, we deduced that there is complete decomposition of the Ni(OH)_2_ precursor into NiO after calcination. Moreover, the XRD peaks were relatively broad, indicating that the NiO crystallites were in the range of the nanometre scale^40^. In Fig. 8, the flower-like Ni-PTA catalyst data shows an obvious diffraction peak around 2θ = 20° from the NiO, and some inconspicuous peaks of PTA crystal (11°, 26°, and 34°, JCPDS No. 50–0657) suggesting that the attached PTA molecule has monolayer dispersion on the surface of flower-like NiO^41^.

The Hydrogen temperature-programmed reduction (H_2_-TPR) profiles of the flower-like NiO and the flower-like Ni-PTA catalyst are displayed in Fig. 8B. The flower-like NiO exhibited only a single reduction peak at temperatures around 450 °C, corresponding to the reduction of NiO to Ni^42^. The H_2_-TPR pattern of the flower-like Ni-PTA catalyst presented two strong peaks at 516 °C and 574 °C, and also a weak peak at 739 °C. For the PTA loaded flower-like NiO, the first peak of NiO species at 516 °C was shifted towards the higher temperature, caused by the reduction of nickel oxide upon interaction with PTA (this finding will be discussed in more detail with the XPS results below). The second peak was around 500 °C for the flower-like Ni-PTA catalyst, and was assigned to the deprotonation of the acid with concurrent nonreductive loss of lattice oxygen^43^. It has been reported that the PTA hydrate began to decompose at 470 °C by release of 1.5 H_2_O, and its thermal decomposition was complete above 610 °C^44^. The weak peak appeared at a temperature higher than the PTA decomposition temperature, probably due to extensive reduction of the constituent oxides of the decomposed acid^45^.

**Figure 9:**
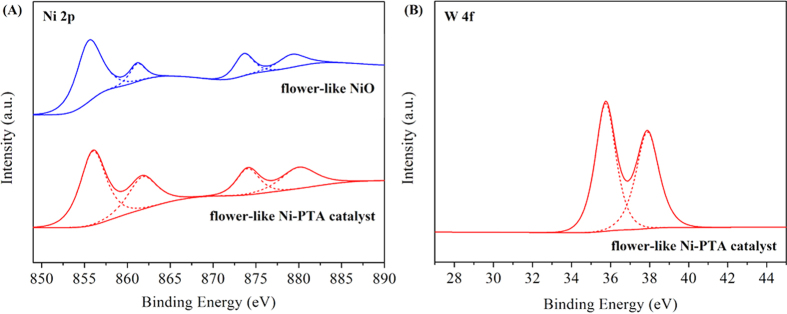
XPS spectra of (**A**) Ni 2p and (**B**) W 4f levels of the synthetic flower-like NiO and flower-like Ni-PTA catalyst.

XPS is a reliable method for investigating the oxidation state of atoms with partially filled valence bonds in the top few layers of material surfaces^46^. Figure 9 exhibits the XPS results of the synthetic flower-like NiO and the flower-like Ni-PTA catalyst, where the peaks of Ni 2p and W 4f were clearly observed. The Ni 2p spectrum of the flower-like NiO (Fig. 9A) showed double peaks for the 2p_3/2_ and 2p_1/2_ components at 855.49 eV and 873.58 eV, characteristic of the NiO phase and in accordance with previous literature^47^. In comparison with the standard binding energy (BE) of pure NiO (854.2 eV), the BE of oxidized Ni shifted positively by 1.29 eV due to the oxygen vacancy existing on the surface^48^. Furthermore, the presence of two shakeup satellite peaks (Ni 2p_3/2_, satellite: 861.16 eV; Ni 2p_1/2_, satellite: 879.16 eV) indicated the electronic state of the Ni^2+^ ion^49^. Thus the Ni 2p signal can be resolved into four peaks. The first prominent peak was an indication of Ni in oxidized form; the other three small peaks were associated with the several oxo-Ni species, i.e. Ni(OH)_2_ and NiOOH phase^50^,^51^. This observation confirmed that the flower-like Ni-PTA catalyst contained more oxo-Ni species due to the larger peak areas.

The XPS for flower-like Ni-PTA catalyst clearly showed the existence of assembled PTA on the flower-like NiO (Fig. 9B), as evidenced by the detection of W. Generally, the W 4f spectrum of bulk PTA shows W 4f_7/2_ at 35.8 eV^52^. In Fig. 9B, the peaks at 35.78 and 37.89 eV could be assigned to W 4f_7/2_ and W 4f_5/2_, respectively, characteristic of the W^6+^ ion^53^. However, the peak at 36.5 eV has previously been ascribed to W^6+ ^54. Such a negative shift could be due to the rich electron density on the tungsten atoms arising from the Ni-O-W linkage in the catalyst^55^. The shift in BE of the W 4f states are ascribed to the reduction of tungsten ions, while the positive shifts in binding energies of the Ni 2p states are ascribed to the oxidation of nickel ions. This implies that Ni species might donate partial electrons to W oxide species, due to the presence of the PTA. These XPS results demonstrate the successful preparation of the flower-like Ni-PTA catalyst.

In addition, the surface atomic contents of the flower-like Ni-PTA catalyst were 19.02% for Ni and 3.45% for W, with an atomic ratio of 5.51:1, indicating that the catalyst was comprised of more nickel atoms and was deficient in tungsten atoms on the surface. These values are consistent with SEM-EDX mapping analysis, and are lower than that of the stoichiometric flower-like NiO because of the resulting decrease in Ni atoms due to the addition of PTA.

### Catalytic Properties of the flower-like Ni-PTA catalyst in hydrotreatment of Jatropha oil

**Figure 10:**
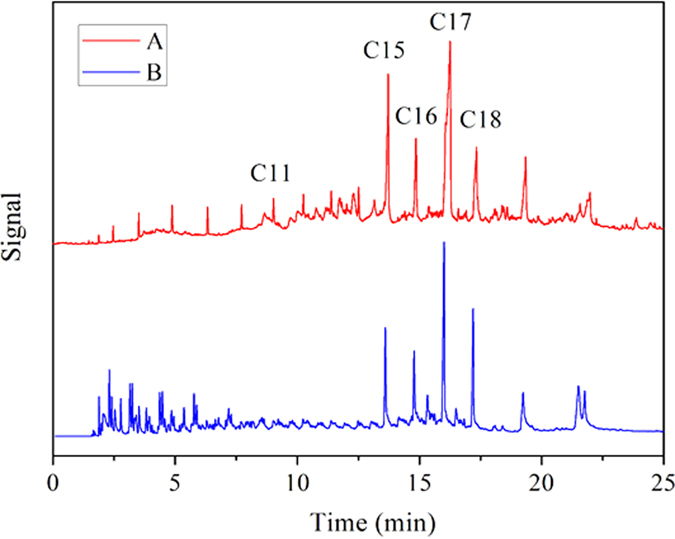
GC charts of product oil from hydrotreatment of Jatropha oil over flower-like Ni-PTA catalyst (**A**) and Ni-PTA/Al_2_O_3_ catalyst (**B**).

Figure 10 shows the GC charts of liquid product oil from the hydrotreatment of Jatropha oil using the flower-like Ni-PTA catalyst (A) and Ni-PTA/Al_2_O_3_ catalyst (B) at 360 °C, 3 MPa. Two tested catalysts exhibited a good hydrogenation activity, and C15H32, C16H34, C17H36, and C18H38 were the main products. However, the cracking activity of the Ni-PTA/Al_2_O_3_ catalyst was higher under the experimental conditions, with more short chain liquid hydrocarbons (<C11), as seen in Fig. 10B. The heavy fraction (>C18) was observed in both catalysts, and mainly contained long chain esters formed by esterification of alcohol and free fatty acid^56^,^57^.

Table 1 shows the conversion of Jatropha oil and the product selectivity using the flower-like Ni-PTA catalyst and the Ni-PTA/Al_2_O_3_ catalyst in the hydroprocessing of Jatropha oil. The conversion of Jatropha oil and C11-C18 selectivity over the Ni-PTA/Al_2_O_3_ catalyst was lower than that for the flower-like Ni-PTA catalyst. This was due to the considerable gasoline fraction (<C11). We saw from the (C15 + C17)/(C16 + C18) ratio that the predominant oxygen removal pathways are similar. The results indicated that both of the decarboxylation + decarbonylation routes (which forms C15 and C17) are much more favored than the hydrodeoxygenation route (which forms C16 and C18)^58^.

Although the conversion of the flower-like Ni-PTA was close to that of the Ni-PTA/Al_2_O_3_, the morphology and structure were very different. The BET surface area for Ni-PTA/Al_2_O_3_ was 250 m^2^/g, and for the flower-like Ni-PTA it was only 86 m^2^/g. Generally, the nanosize scale and high surface area should allow the catalysts to exhibit higher activity^16^. However, surface area is not the only factor that determines the activity of the catalyst. The Ni-PTA catalyst with flower-like morphology possessed more metal atoms on edges and corners (see TEM and EDX), which are the active sites for adsorption of Jatropha oil. Additionally, mesoporous surfaces can promote the desorption of the products, following by the inhibition of some side reactions^59^, consistent with the SEM and BET result.

In this experiment, 10 g of the Ni-PTA/Al_2_O_3_ catalyst was required to reach the same catalytic activity (conversion and selectivity) as only 0.6 g of the flower-like Ni-PTA catalyst. Compared with the Ni-PTA/Al_2_O_3_ catalyst, the catalytic efficiency was increased more than 15-fold by the flower-like Ni-PTA catalyst. There is no support in the flower-like Ni-PTA catalyst, and the Ni content per unit of area in the flower-like Ni-PTA catalyst (19.02%) was much higher than that in the Ni-PTA/Al_2_O_3_ catalyst (5.74%), revealed by XPS analysis. This likely led to the formation of more activity sites (metal and acid center) which would improve the catalytic activity. Figure S1 (see supplementary information) shows the GC chart of product oil from the hydrotreatment experiment over the Ni-PTA/Al_2_O_3_ catalyst with the same mass (0.6 g) as the flower-like Ni-PTA catalyst. Although the GC chart was similar to that shown in Fig. 10, Jatropha oil conversion was 85.31% and C11-C18 selectivity was only 28.48%. Therefore, its catalytic activity was much lower than that of the flower-like Ni-PTA catalyst with the same mass. Thus, the morphology and flower-like architectures are important for efficient catalytic hydrogenation.

To investigate the stability of the catalyst, long term experiments of 100 h were conducted at 360 °C, 3 MPa, 15 h^−1^. As seen in Figure S2, the catalyst did not show deactivation during 100 h of on-stream reaction. The conversion of Jatropha oil and the selectivity of product C11–C18 remained constant, illustrating the good stability of the flower-like Ni-PTA catalyst in the hydrotreatment of Jatropha oil. As shown in Figure S3A, the flower-like architectures of the spent flower-like Ni-PTA catalyst was slightly changed and the size was significantly aggregated and sintered to 1–2 μm after the reaction, as detected by SEM. The TGA analysis of the spent catalysts is shown in Figure S3B. According to the reference of ISO 6964–1986, the decoking of carbon deposited on the catalyst surface usually occurred at over 550 °C in an oxidative environment^60^. The cumulative value of the weight loss occurred above 550 °C was used to estimate the coke formed on the catalyst in this work, where the carbon residue after the reaction increased 2.56%. The low coke formation in the flower-like Ni-PTA catalyst is likely due to the the space constraint in medium size pores for the formation of bulky coke precursors^61^.

## Conclusion

Green diesel (renewable liquid alkanes) can be produced from hydrotreatment of plant oil over a flower-like Ni-PTA catalyst without sulfuration. This novel catalyst was prepared with a flower-like nickel oxide (NiO) supported phosphotungstic acid (PTA). This catalyst was tested with Jatropha oil, and the performance was evaluated by GC, showing conversion of 98.95% and C11-C18 selectivity of 70.93% at 360 °C, 3 MPa, 15 h^−1^. The flower-like Ni-PTA catalyst showed good stability up to 100 h and the carbon residue of the spent catalyst increased 2.56%. Compared to the Ni-PTA/Al_2_O_3_ catalyst, the necessary catalyst mass was reduced from 10 to 0.6 g, and the catalytic efficiency of the flower-like Ni-PTA catalyst increased 16 to 17-fold. The experimental results also showed that reaction temperature, time, and the presence of SDS play critical roles in the formation of flower-like Ni(OH)_2_. The morphology evolution was found to be a two-stage growth process. The non-sulfided flower-like Ni-PTA catalyst removes the need for a presulfurization step, avoiding the harm of sulfide to the environment and to human health. In conclusion, this new catalyst has great potential as an alternative to sulfided catalysts for green diesel production.

## Additional Information

**How to cite this article**: Liu, J. *et al.* A Non-sulfided flower-like Ni-PTA Catalyst that Enhances the Hydrotreatment Efficiency of Plant Oil to Produce Green Diesel. *Sci. Rep.* **5**, 15576; doi: 10.1038/srep15576 (2015).

## Supplementary Material

Supplementary Information

## Figures and Tables

**Table 1:** Hydrotreatment of Jatropha oil using the Ni-PTA/Al_2_O_3_ catalyst and the flower-like Ni-PTA catalyst at 360 °C, 3 MPa.

Catalyst	flower-like Ni-PTA catalyst	Ni-PTA/Al_2_O_3_ catalyst
Conversion (%)	98.95	95.13
<C11 selectivity (%)	10.08	39.40
C11–C18 selectivity (%)	70.93	45.60
>C18 selectivity (%)	18.99	15.00
(C15 + C17)/(C16 + C18) ratio	2.63	1.70
Iso/n ratio	0.76	1.09
