# Supplementary material for: A Non-sulfided flower-like Ni-PTA Catalyst that Enhances the Hydrotreatment Efficiency of Plant Oil to Produce Green Diesel
Source: Sci Rep. 2015 Oct 27;5:15576. doi: 10.1038/srep15576 (PMC4622087; doi:10.1038/srep15576)
Supplement: Supplementary Information [file srep15576-s1.doc]

**Supplementary Information**

**A Non-sulfided flower-like Ni-PTA Catalyst that Enhances the** **Hydrotreatment Efficiency of Plant Oil to Produce Green Diesel**

Jing Liu ^1^, Pan Chen ^1^, Lihong Deng ^1^, Jing He ^1^, Luying Wang ^1^, Long Rong ^2^, Jiandu Lei ^1^,*

^1^ *Beijing Key Laboratory of Lignocellulosic Chemistry*, Beijing Forestry University, Beijing 100083, P. R. China

^2^ Key Laboratory for Biomechanics and Mechanobiology of Ministry of Education, School of Biological Science and Medical Engineering, Beihang University, Beijing 100191, P. R. China


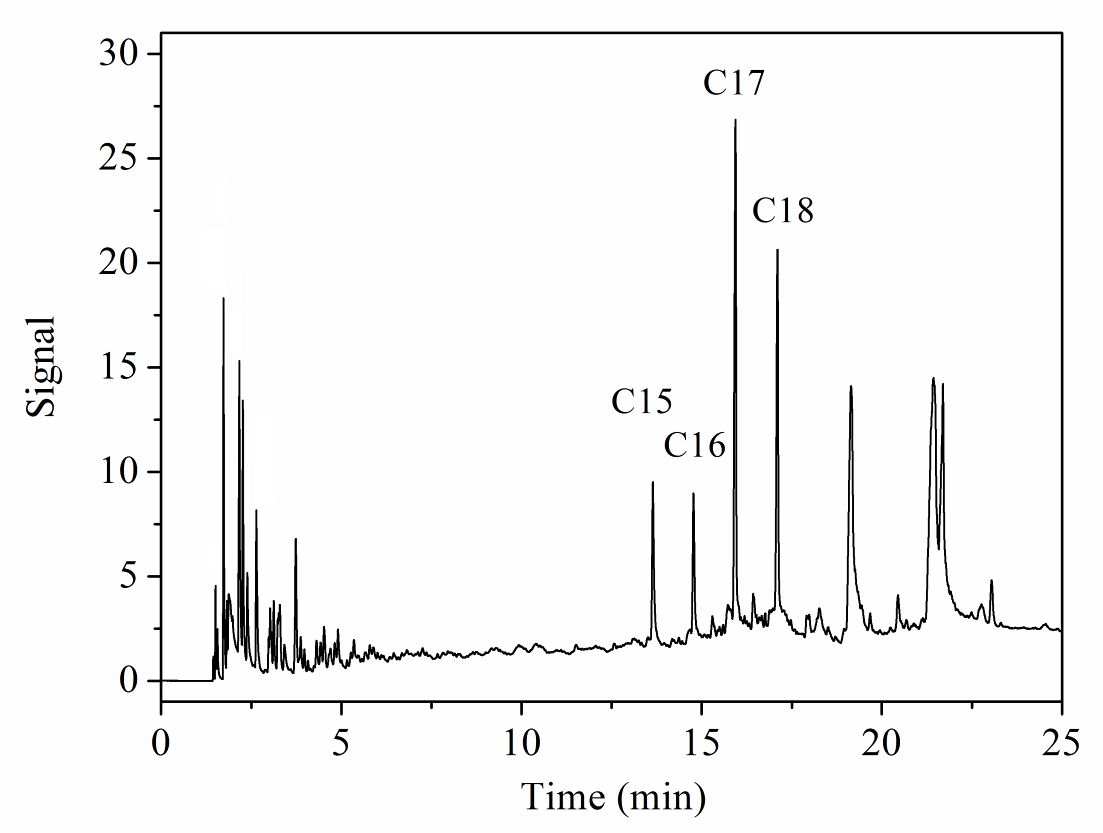


Figure S1 GC charts of product oil from hydrotreatment of Jatropha oil over 0.6 g of Ni-PTA/Al_2_O_3_ catalyst.


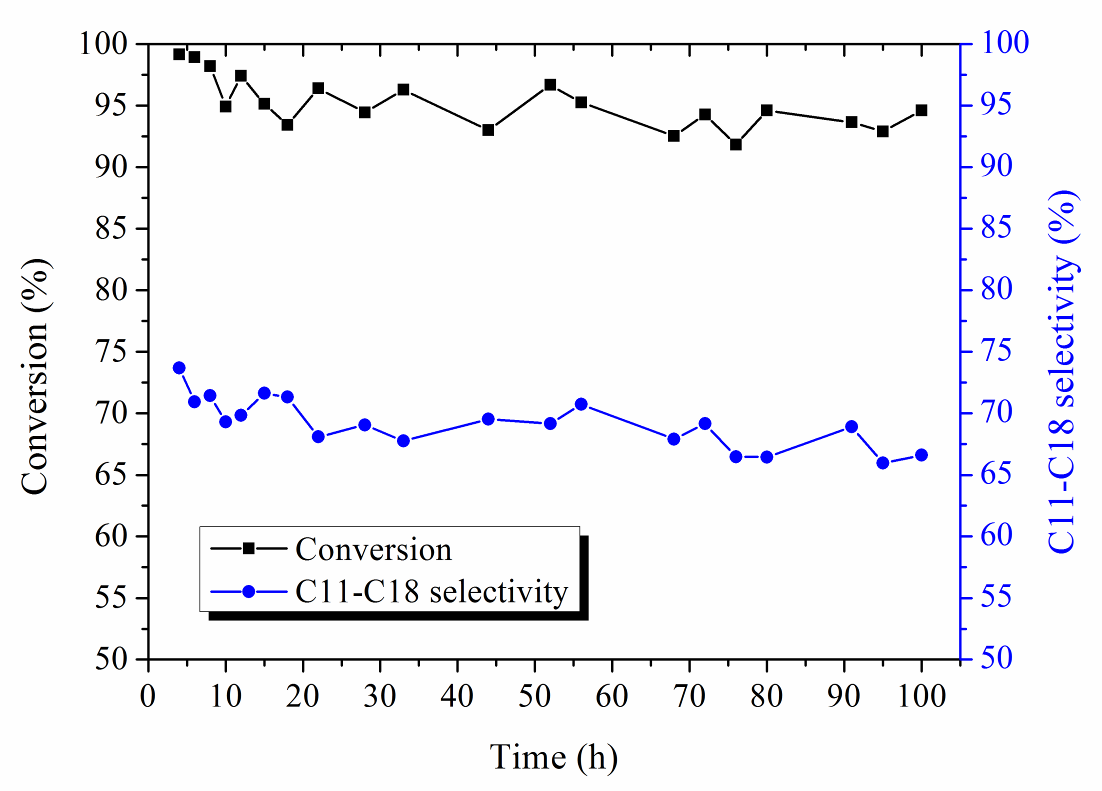


Figure S2 Conversion of Jatropha oil and product C11-C18 selectivity during a long-term stability test (360 °C, 3 MPa, 15 h^−1^).


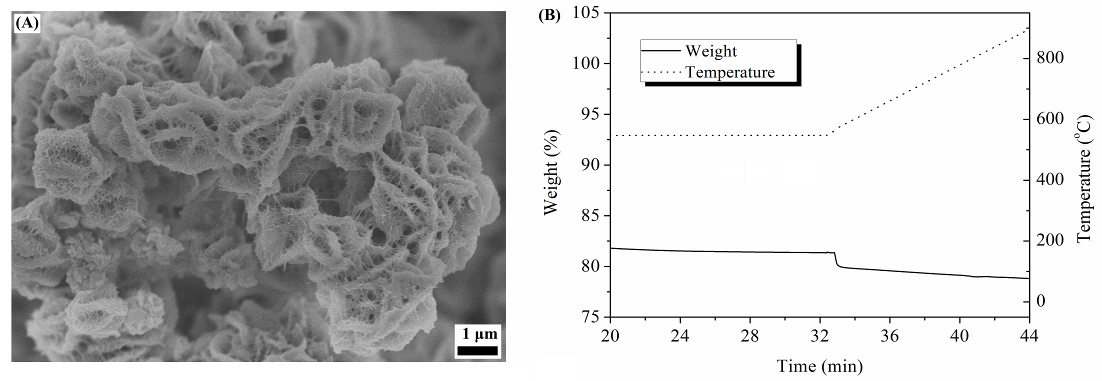


Figure S3 SEM image (A) and TGA curve (B) of spent flower-like Ni-PTA catalyst.
